# Autonomous Sensors Powered by Energy Harvesting from von Karman Vortices in Airflow

**DOI:** 10.3390/s17092100

**Published:** 2017-09-13

**Authors:** Marco Demori, Marco Ferrari, Arianna Bonzanini, Pietro Poesio, Vittorio Ferrari

**Affiliations:** 1 Department of Information Engineering, University of Brescia, Via Branze 38, 25123 Brescia, Italy; marco.ferrari@unibs.it (M.F.); vittorio.ferrari@unibs.it (V.F.); 2 Department of Mechanical and Industrial Engineering, University of Brescia, Via Branze 38, 25123 Brescia, Italy; a.bonzanini001@unibs.it (A.B.); pietro.poesio@unibs.it (P.P.)

**Keywords:** autonomous sensors, flow induced vibrations, piezoelectric energy harvesting, von Karman vortices

## Abstract

In this paper an energy harvesting system based on a piezoelectric converter to extract energy from airflow and use it to power battery-less sensors is presented. The converter is embedded as a part of a flexure beam that is put into vibrations by von Karman vortices detached from a bluff body placed upstream. The vortex street has been investigated by Computational Fluid Dynamics (CFD) simulations, aiming at assessing the vortex shedding frequency as a function of the flow velocity. From the simulation results the preferred positioning of the beam behind the bluff body has been derived. In the experimental characterization the electrical output from the converter has been measured for different flow velocities and beam orientations. Highest conversion effectiveness is obtained by an optimal orientation of the beam, to exploit the maximum forcing, and for flow velocities where the repetition frequency of the vortices allows to excite the beam resonant frequency at its first flexural mode. The possibility to power battery-less sensors and make them autonomous has been shown by developing an energy management and signal conditioning electronic circuit plus two sensors for measuring temperature and flow velocity and transmitting their values over a RF signal. A harvested power of about 650 μW with retransmission intervals below 2 min have been obtained for the optimal flow velocity of 4 m/s.

## 1. Introduction

Energy harvesting from von Karman vortices can prove effective since vortices behind obstacles in a flow represent an ideal forcing for a vibrating energy harvester. The vortices are obtained from a conversion of a unidirectional flow into an alternate velocity field [1,2,3,4,5,6].

Energy harvesting from von Karman vortices has been studied in recent years to demonstrate and optimize this possibility. Various configurations and energy converters from flows in different fluids, air or liquid, have been investigated. In several works, flexible piezoelectric membranes in the wake of a bluff body were used as the converter. In particular, it was demonstrated that the optimal condition to harvest energy is obtained when the shedding frequency of the vortices matches the fundamental frequency of the membrane [7]. The flapping dynamics of a piezoelectric membrane in a wake of a square cylinder were studied in [8,9], in terms of flapping modes, amplitude, frequency, and strain distribution along the membrane length. Different bluff body shapes, such as cylinder or parallelepiped with triangular section, have been extensively investigated demonstrating good efficacy in the generation of vortices [7,8,10,11,12]. The oscillations and the energy harvesting effectiveness of a piezoelectric beam element in the wake of a cylinder were extensively investigated in [10,13]. The best performances were obtained when the vortices induce resonance in a beam placed at an optimal positioning, i.e., with the tip of the beam at a distance of about two cylinder diameters downstream of the cylinder in the center line. The possibility to improve the harvested energy by exploiting the proximity effects of multiple vibrating harvesters has been investigated in [3,14]. An example of energy harvesting from von Karman vortices in a liquid water flow was presented in [15], where pressure variations were used to generate a periodic displacement of a magnet connected to a membrane as a part of the pipe wall. The produced movement of the magnet was exploited to generate electrical power by means of the relative motion between the magnet and a coil. A wide review of energy harvesting devices that exploit flow induced vibrations and of their possible application on aerospace vehicles was presented in [16]. Proof-of-concept preliminary experiments of autonomous sensors powered by energy harvesting from vortices in airflow were presented in [17].

This paper innovatively presents the design, analysis, and experimental validation of a complete energy harvesting system that exploits von Karman vortices. The energy harvester is composed of a bluff body and a flexure beam with an embedded piezoelectric converter. The beam is placed behind the bluff body to collect the alternate forcing in the vortex street. The analysis has focused on the beam orientations, to exploit the maximum alternate forcing, and the conversion effectiveness as a function of the flow velocity, i.e., the repetition frequency of the vortices and the magnitude of the alternate forcing. The aim of this work is to demonstrate the possibility to successfully power autonomous, i.e., battery-less, sensors that measure the flow properties and send data over a wireless Radio-Frequency (RF) link. The proposed configuration can be used, for example, to realize self-powered Wireless Sensor Networks (WSNs), and several applications may be identified, for instance, in the environmental monitoring or structural health monitoring in critical positions where batteries are difficult to replace, in the general framework of the Internet of Things (IoT) scenario.

The energy harvester has been mounted in a wind tunnel system developed to obtain two specific possibilities, namely rotate the beam at different orientation angles with respect to the prevailing flow direction, and generate different flow velocities in the wind tunnel. In this way, the harvesting performances could be investigated as a function of the above two parameters. In fact, on the one hand, the orientation angle affects the interaction between the beam and the forcing pressure field. On the other hand, the repetition frequency of the vortices and the coupling with the beam mechanical resonance depend on the flow velocity. In order to perform the proposed investigations, initially the voltage from the converter has been measured and analyzed in both time and frequency domains. The harvesting performances have been first quantified by measuring the harvested power on a resistive load as a function of the flow velocity. Then the possibility to exploit the harvested energy to power autonomous sensors has been investigated. To this purpose, the converter has been connected to a tailored battery-less electronic circuit that accumulates the harvested energy and powers two sensors and a RF transmitter. When enough accumulated energy is available, the circuit triggers a measurement-plus-transmission cycle. Therefore, the system operates intermittently with repeated transmissions of a RF signal carrying data from the sensors from which information on the flow properties can be extracted.

## 2. Energy Harvesting System

### 2.1. System Description

**Figure 1:**
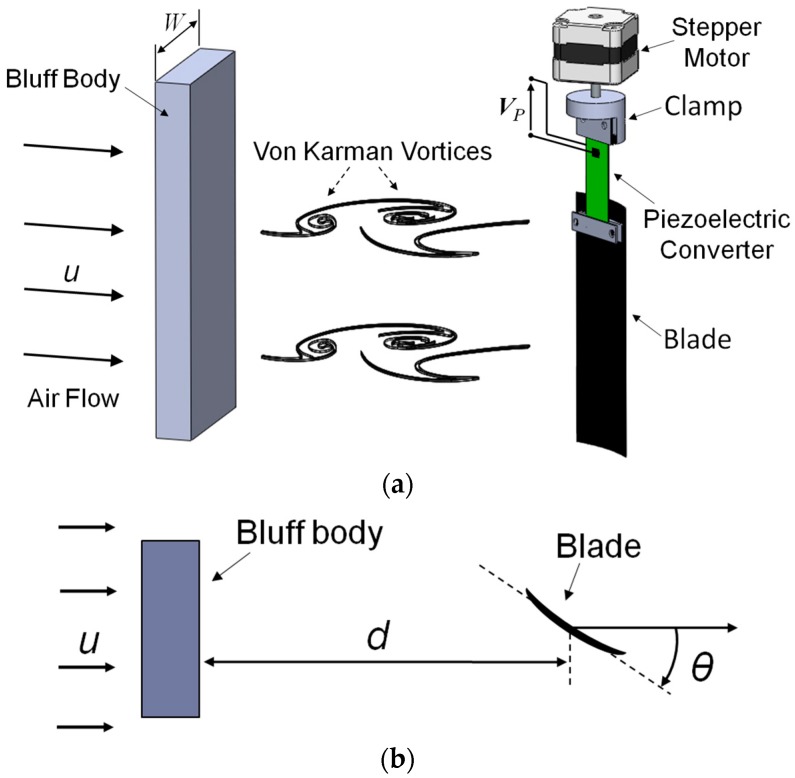
Sketch of the system: the bluff body is placed perpendicular to the flow direction and it is *W* = 55 mm wide and 20 mm thick; the beam is composed of the piezoelectric converter, with 45 mm length and 20 mm width, and the blade with the air-foil profile cross-section, with 70 mm length and 30 mm width (**a**); Top view shows the position of the blade at *d* from the bluff body, and the orientation angle *θ* (**b**).

As shown in Figure 1, a rectangular prism, with a width *W*, is placed in the airflow field acting as bluff body for the generation of the vortices. The rectangular cross-section for the bluff body has been chosen as an effective shape for the generation of von Karman vortices [8]. In addition, it represents a sufficiently generic obstacle, yet no optimization of the bluff body shape was considered within the scope of this work. The beam is placed in the vortex street behind the obstacle in order to undergo periodic excitation. The beam is composed of a bimorph piezoelectric converter (WAC3X/18) and of a blade profile with air-foil profile cross-section connected to the free end of the converter. The converter consists of a flat steel plate with lead zirconate titanate (PZT) films and electrode metal layers on both the surfaces [18]. Both the PZT films have a thickness of about 200 μm. The converter is clamped at one end to obtain a cantilever structure and it represents the flexural part of the beam, where the elastic response is concentrated. The blade is connected to the free end of the converter in order to increase coupling with the pressure filed generated by the flow in the vortex street. The blade is made of a rigid plastic which has an asymmetric air-foil profile cross-section.

The bluff body and the beam are mounted in a low-velocity wind tunnel creating a compact characterization system. The beam has been placed at a properly selected distance *d* behind the bluff body, in the center line, along which the maximum excitation of the beam is expected. The beam is connected to the shaft of a stepper motor by means of a fit-for-purpose clamp in order to vary the orientation of the blade surface with respect to the flow direction. The orientation angle *θ* is defined as the angle between the direction normal to the bluff body width *W* and the beam face, i.e., the plane of the blade.

### 2.2. Flow Simulations

An investigation of the von Karman street behind the proposed bluff body has been performed by Computational Fluid Dynamics (CFD) simulations. Before the subsequent experimental analysis, simulations have been carried out to obtain an estimation of the shedding frequency *f_u_* of the vortices as a function of the inlet flow velocity. The simulation analysis was intentionally performed without the beam placed in the flow, since a detailed description of the system with the beam requires a fluid-structure interaction study, which complexity is out of the scope of this paper. Despite the simplified approach, the CFD simulations retain full validity and substantial importance since neither the shedding frequency nor the von Karman street are influenced to a significant extent by the beam presence. Simulations have been performed on the two-dimensional middle horizontal cross-section of the wind tunnel. These simulations are representative of the investigated flow, since the turbulence allows neglecting the effect of the upper and bottom wind tunnel walls, which is confined within the boundary layer.

The large time-step transient solver pimpleFoam of the free software OpenFOAM, version 16.06, has been used to solve an incompressible flow using the k-ε turbulence model. The default model constants available in OpenFOAM have been employed together with the kqRWallFunction and epsilonWallFunction to describe k and ε at walls. The simulated cross-section has a width of 17.5 cm (about 3.5 *W*) and a length of 62 cm. The bluff body has been placed 25 cm after the inlet section. The inlet flow velocity *u* has been varied in the range between 2.0 and 7.0 m/s, which corresponds to the actual working condition of the wind tunnel. The frequency of the vortices has been calculated from the time evolution of the simulated velocity field observed at 90 mm downstream from the bluff body.

A grid refinement analysis has been performed to assess the convergence of the simulation results. The vortex shedding frequency *f_u_* has been adopted as the reference quantity to assess the grid convergence. The Grid Convergence Index (GCI) method, proposed by Roache [19], has been adopted. Three different structured grids have been employed: the coarsest one has a grid spacing of *h*_3_ = 4 mm, and the refinement ratio *r* = *h*_3_/*h*_2_ = *h*_2_/*h*_1_ = $\sqrt{2}$ has been used to obtain a doubling of the cells number between successive grid steps. The maximum Courant number (CFL condition) has been set as unitary in all the simulations and the time step has been computed consequently. The analysis has been carried out for two different inlet flow velocities of 2.0 m/s and 7.0 m/s which respectively represent the minimum and the maximum values. This is because if the grid convergence is observed at the boundaries of the range of interest, then it is ensured within the whole range. Table 1 reports the vortex frequency *f_u_* computed for the three different grids. From these values the order of convergence *p*, the GCI_2,3_ between the medium and the coarse grids, and the GCI_1,2_ between the fine and the medium grids have been computed; a safety factor of 3 has been employed in computing the GCI, as suggested in [19]. The last column of Table 1 reports the relation between the GCIs, adopted to observe if the three checked grids are in the asymptotic range of convergence. Since the order of convergence *p* is about 3 and the GCIs report a low uncertainty on both the two couples of grids and the uncertainty is reduced as the grid is refined, then grid convergence is ensured. Moreover, the relation between the GCIs is near unity, which ensures the analyzed grids are within the asymptotic range of convergence. By the grid analysis results, the finest grid *h*_1_ = 2 mm has been adopted to perform all the simulations.

**Figure 2:**
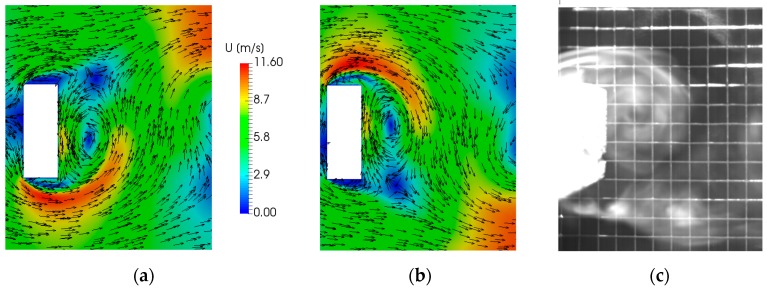
Simulated velocity field of two subsequent vortices (**a**,**b**); Comparison between the smoke visualization image of the air streamline in the vortex region (**c**) and the flow simulation (**b**). The 1-cm-pitch wire-mesh shown in (**c**) has been placed outside the wind tunnel as dimensional reference and it is not interfering with the flow.

Figure 2a,b show the simulated flow velocity field in the vortex street correspondent to the generation of two subsequent vortices at the left and right edge of the bluff body, respectively. In addition, Figure 2c shows for comparison the smoke visualization image of the air streamline in the vortex region. A good agreement in the dimension and shape of the vortices can be observed.

**Figure 3:**
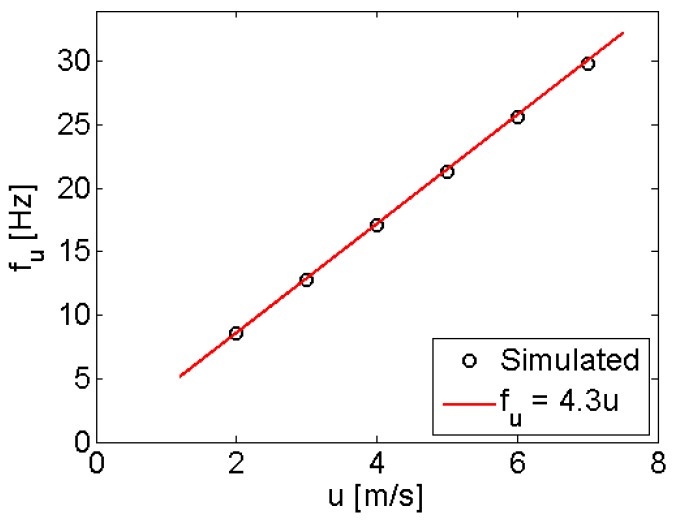
Vortices shedding frequency as function of the inlet flow velocity obtained by CFD simulations. The expected linear trend is observed.

The flow in the vortex street consists of a periodic sequence of alternate vortices. Figure 3 reports the simulated shedding frequency *f_u_* of the vortices, as function of the inlet flow velocity. Results suggest linear relation between *u* and *f_u_*, in good agreement with the theoretical expectations since the shedding frequency *f_u_* of the vortices behind a bluff body with a characteristic dimension *W*, is linked to the inlet flow velocity *u* by:

(1) $$f_{u}=\frac{St\cdot u}{W}$$

where *St* is the Strouhal number [10,12,15,20] of the flow. From the linear interpolation of the *f_u_* values, shown in Figure 3, a Strouhal number *St_sim_* ≈ 0.23 has been obtained from the CFD simulations.

**Figure 4:**
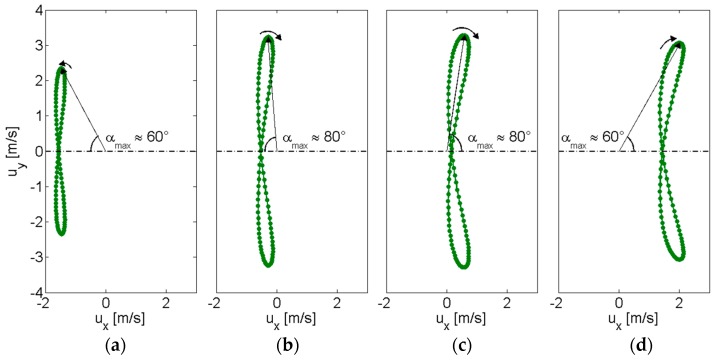
Time evolution of the velocity vector obtained by the components *u_y_* vs. *u_x_* at 30 mm (**a**); 70 mm (**b**); 90 mm (**c**); 130 mm (**d**) behind the bluff body, in the center line, for a complete cycle composed of two subsequent vortices. The trajectory of the velocity vector tip is reported in green and the vector correspondent to the maximum angle α_max_ between the central axis and the velocity vector is shown.

Besides a prediction of the working frequency range of the oscillating beam placed in the vortex street, numerical simulations can provide information on the preferred position behind the bluff body where the beam should be placed. The velocity components, *u_x_* and *u_y_* in the streamwise and spanwise directions, respectively, have been obtained by simulations at different positions behind the bluff body, namely 30, 70, 90, and 130 mm along the central axis. The inlet flow velocity of 5 m/s has been considered in all cases. The plots of Figure 4 report the loci of the velocity vectors obtained from the components *u_x_* and *u_y_* on the *x*-axis and on the *y*-axis, respectively, during a cycle composed by two subsequent vortices. Thus, the time evolution of the velocity vector magnitude $\left|u\right|=\;{\left(u_{x}+\;u_{y}\right)}^{1/2}$ and angle α between the central axis and the vector direction can be compared in the four positions.

For the two intermediate distances 70 mm (Figure 4b), and 90 mm (Figure 4c), both the velocity magnitude |u| and the angle α present the maximum variations during the complete cycle, if compared to the other positions 30 mm (Figure 4a), and 130 mm (Figure 4d). In fact, together with the maximum variations of α in Figure 4b,c, which are about −80° < α < +80°, the velocity magnitude spans between 0.5 ÷ 3.3 m/s and 0.1 ÷ 3.4 m/s, respectively. Instead, at short distance behind the bluff body (Figure 4a), not only the magnitude variation, 1.6 ÷ 2.8 m/s, is lower, but also the same is true for the angle variation, namely about −60° < α < +60°. At this position a recirculation region is present behind the bluff body, as it can be observed by the negative value of *u_x_*. As the distance from the bluff body increases (Figure 4d, both |u| and α variations decrease to 1.5 ÷ 3.7 m/s and −60° < α < +60°, respectively, since *u_x_* approaches the inlet flow velocity and *u_y_* reduces because of turbulent dissipation. The results suggest that the distance range between 70 and 90 mm are expected to provide an effective alternate forcing for a vibrating energy harvester due to the best combination between the magnitude variation and the angle variation taking place.

Following these considerations, the beam has been placed at *d* = 80 mm ≈ 1.5 *W* downstream from the bluff body. This choice is supported also by the results reported in [10], since 1.5 *W* represents a trade-off between the distance of two times the bluff body width, 2 *W*, and the distance of two hydraulic diameters of the rectangular section of the bluff body, 1.1 *W*.

### 2.3. Experimental Characterization

**Figure 5:**
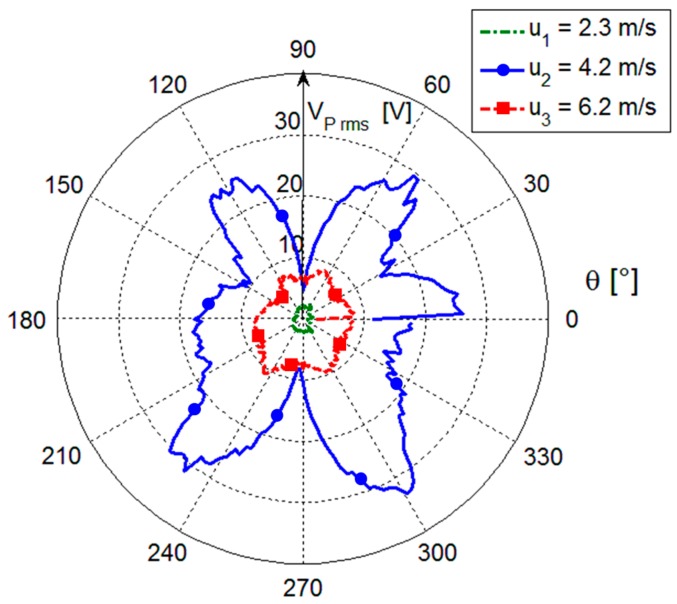
Polar visualization of the rms value of the *V_P_* measurements as a function of *θ* for the three different flow velocities *u*_1_, *u*_2_ and *u*_3_. An accuracy of ±1.8° can be assumed in the orientations *θ* that corresponds to an uncertainty of one step of the stepper motor.

The converter open-circuit voltage *V_P_*, which is associated to the beam oscillations, has been measured for different beam orientations at three different flow velocities generated in the wind tunnel. The whole range of orientations of 360° has been divided in 200 steps leading to an angle resolution of 1.8°. For each step the voltage *V_P_* has been measured by using an MSO-X 3014A digital oscilloscope (Agilent, Santa Clara, CA, USA) with an input impedance of 10 MΩ in parallel with 15 pF that overall can be assumed as an open circuit load for the adopted piezoelectric converter that has an internal equivalent impedance composed of a capacitance *C_P_* ≈ 305 nF and a parallel resistor *R_P_* ≈ 250 kΩ as measured at 100 Hz by a HP4194 impedance analyzer (Hewlett-Packard, Palo Alto, CA, USA). The flow velocity has been measured by a 405-V1 hot wire anemometer (Testo SE & Co. KGaA, Lenzkirch, Germany) placed before the bluff body in the center of the initial section of the wind tunnel. The sensor has a velocity resolution of 0.1 m/s and an accuracy of ±0.3 m/s. In Figure 5 the rms values of *V_P_* measured on a time window of 20 s are shown as a function of the angle *θ* for three values of flow velocity, namely *u*_1_ = 2.3 m/s, *u*_2_ = 4.2 m/s and *u*_3_ = 6.2 m/s. As it can be observed, higher rms values are obtained when the orientation angles are about *θ* = 60°, 120°, 240° and 300°. This means that, for such orientations, the beam experiences a better coupling with the forcing pressure field. The slight deviations from perfect symmetry in specular orientations of the beam can be ascribed to the asymmetry of the blade profile.

Moreover, in Figure 5 it can be observed that the highest rms values of *V_P_*, of up to about 30 V, have been achieved for the intermediate velocity *u*_2_*,* with respect to the values below 10 V obtained for the other velocities. This would seem in contrast with the increasing flow intensity and turbulence with velocity progressively increasing from *u*_1_ to *u*_3_. The reason for the highest rms values of *V_P_* at the intermediate velocity *u*_2_ can be found in the repetition frequency of the vortices that at *u*_2_ becomes close to the resonant frequency *f_m_* of the beam first flexural mode, which is of about 13.6 Hz. As a consequence, under this condition, the vortices excite the beam generating larger oscillations compared to other flow velocities. In particular, this happens for *u*_3_, where, despite the higher velocity compared to *u*_2_, lower rms values are generated.

**Figure 6:**
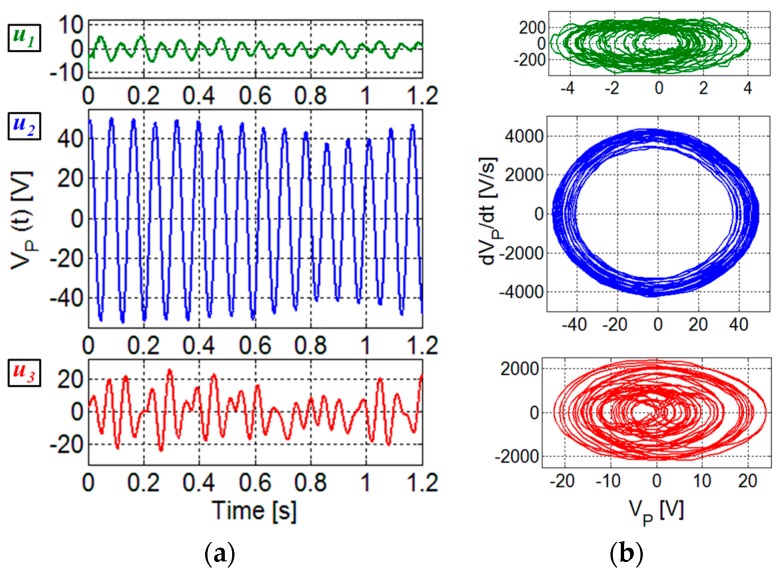
Voltages *V_P_* measured versus time (**a**) and phase planes (**b**) at different flow velocities: *u*_1_ = 2.3 m/s, *u*_2_ = 4.2 m/s, and *u*_3_ = 6.2 m/s for the same orientation angle *θ* = 300°.

The effect of the vortex repetition frequency has been investigated by the analysis of the time and frequency behavior of the measured *V_P_* at the different flow velocities. Figure 6a shows the comparison of the voltages *V_P_* versus time for the three velocities *u*_1_, *u*_2_ and *u*_3_, obtained at the fixed orientation of *θ* = 300°. This comparison confirms that the higher magnitude of *V_P_* associated to the larger oscillations is obtained for the intermediate velocity, since the repetition frequency of the vortices excites the beam resonance. The trajectories in the phase plane [21] derived from the time records of *V_P_* are also reported in Figure 6b. For the piezoelectric element, the open-circuit output voltage *V_P_* and its time derivative d*V_P_*/d*t* are proportional to displacement and velocity, respectively [22]. The comparison of the phase plots show that quasi-periodic oscillations are obtained at *u*_2_, while for *u*_1_ and *u*_3_ sets of manifold trajectories are evident suggesting a more complex dynamical behavior.

**Figure 7:**
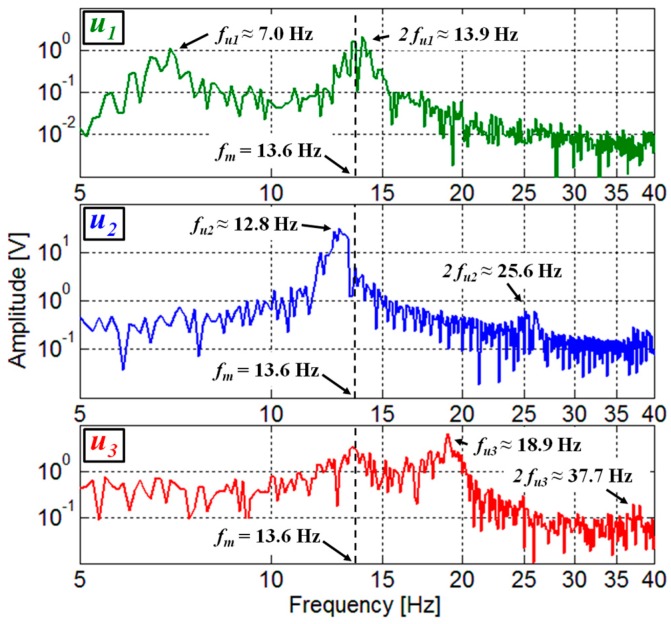
Frequency spectrum of *V_P_* measured for three different flow velocities: *u*_1_ = 2.3 m/s, *u*_2_ = 4.2 m/s, and *u*_3_ = 6.2 m/s for the same orientation angle *θ* = 300°. The indication of frequency of the main components and the resonant frequency of the beam are reported.

The frequency spectra of the measured voltages *V_P_* have been obtained by FFT processing, considering a time window of 20 s. Figure 7 shows the comparison among the frequency spectra of *V_P_* measured for the three different velocities considered in Figure 6. In the spectrum relative to the intermediate velocity *u*_2_, a main component at the corresponding vortex repetition frequency *f_u_*_2_, well beyond the voltage level at other frequencies, is present. Indeed, at the velocity *u*_2_ it was possible to clearly identify *f_u_*_2_ = 12.8 Hz since it is near to the beam resonant frequency of 13.6 Hz. From Equation (1) and the frequency *f_u_*_2_, the Strouhal number of the proposed configuration could thus be determined, resulting in *St* ≈ 0.17; this value is in reasonably good agreement with the value of *St_sim_* ≈ 0.23 obtained by CFD in Section 2.2.

In this way, Equation (1) also allows to calculate the repetition frequencies *f_u_*_1_ and *f_u_*_3_ at *u*_1_ and *u*_3_, resulting in *f_u_*_1_ = 7.0 Hz and *f_u_*_3_ = 18.9 Hz, respectively. As it can be observed in Figure 7, main components in the spectra of *V_P_* for *u*_1_ and *u*_3_ are present at the frequencies *f_u_*_1_ and *f_u_*_3_. This confirms that the oscillations contain a frequency contribution at the repetition frequency of the forcing vortices also for the velocities *u*_1_ and *u*_3_. In addition, it can be observed that in all three spectra components are also present at the second harmonics of the repetition frequencies.

Given the sufficiently high bending stiffness of the piezoelectric element, no significant departure from mechanical linearity is expected in the explored flow range. This is confirmed in Figure 6a where the output voltage *V_P_* is shown to be essentially a sinusoid when the system is excited at a vortex repetition frequency close to the resonant frequency *f*_m_, i.e., for velocity *u*_2_. Then an analysis of the frequency spectra in linear conditions is appropriate.

For this purpose, it can be assumed that the action of a vortex on the beam consists of a force pulse and that the oscillation of the beam caused by the force pulse is a sinusoidal damped oscillation. Thus, when the oscillation is generated by a periodic sequence of vortices with a repetition frequency *f_V_*, the voltage *V_C_*(*t*) at the output of the piezoelectric cantilever, can be expressed as the sum of sinusoidal damped oscillations:

(2) $$V_{C}\left(t\right)=\underset{k}{\sum }s\left(t-kT_{v}\right)A_{d}e^{-\alpha_{d}\left(t-kT_{v}\right)}\cos\left(2\pi f_{d}\left(t-kT_{v}\right)\right)$$

where *s(t)* represents the step function, and *T_v_* = 1/*f_v_* is the time interval between two subsequent vortices, and *k* is an integer. The parameters *A_d_*, *α_d_* and *f_d_* represent, respectively, the amplitude, damping factor and oscillation frequency of the damped oscillation of the beam due to an applied force pulse.

The frequency spectrum ***V_C_****(f)* can be derived by the Fourier transform of the time function in Equation (2) as follows:

(3) $$V_{C}\left(f\right)=\left[A_{d}\frac{1}{\alpha_{d}+j2\pi \left(f-f_{d}\right)}\right]\cdot \frac{1}{T_{v}}\delta_{f_{v}}\left(f\right)$$

**Figure 8:**
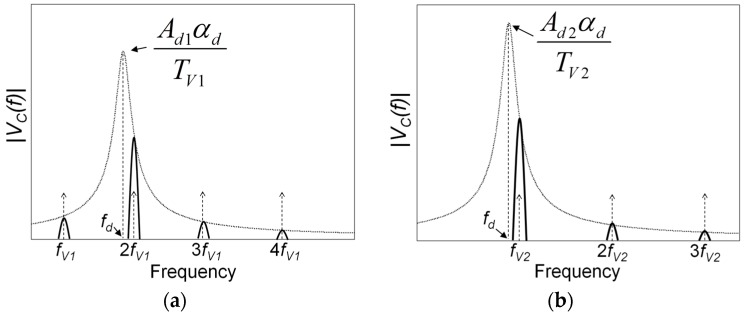
Frequency spectra of the magnitude of the voltage ***V_C_*** calculated by Equation (3). Two cases are considered: repetition frequency of the vortices *f_V_*_1_ below the frequency *f_d_* of the damped oscillation (**a**); repetition frequency of the vortices *f_V_*_2_ above the frequency *f_d_* (**b**).

The spectrum ***V_C_****(f)* consists of the product of two terms, where the first term represents the spectrum of a single damped oscillation of the beam centered at its damped frequency *f_d_*, and the second term *δ_fv_(f)* consists of a sequence of Dirac pulses equally spaced of *f_v_*. A graphical representation of the magnitude of ***V_C_****(f)*, derived from Equation (3), is shown in Figure 8.

The comparison between the spectra of the measured *V_P_* as shown in Figure 7 and the spectra predicted from the present analysis confirms the presence in the experimental results of main components at the repetition frequency of the vortices and at its harmonics. However, unlike an ideal force pulse, the force pulses associated to the vortices have a finite duration in real conditions. This reflects in the spectra of the measured *V_P_* with the presence of pulses with a finite width. In addition, in Figure 8 it can be observed that the components in the region of the damped oscillation frequency *f_d_* are amplified by the resonance effect. This is well confirmed in the spectra of Figure 7 for all the components in the region around *f_m_*. In particular, it is evident for the velocity *u*_1_, where the second harmonic component at 2*f_u_*_1_ is close to *f_m_* and its magnitude exceeds the fundamental component at the vortex repetition frequency *f_u_*_1_.

### 2.4. Harvested Power Measurements

Tests were then performed to measure the power harvested by the converter and delivered to a load in different excitation conditions. A resistive load has been used to evaluate the available active power, and the optimal value of the load resistor has been investigated to obtain the maximum power [23,24,25,26]. For this purpose, the converter was connected to a resistive load to obtain on average the maximum power in the explored frequency range between about 5 and 50 Hz. The range of the optimal load resistor has been calculated through the simplified relation *R_opt_* ≈ 1/(2π*f_m_C_P_*), where *C_P_* is the equivalent internal capacitance of the converter as described in Section 2.1 [24,25]. Then, the specific value of the optimal load resistor *R_L_* = 15 kΩ was determined through an experimental optimization process to obtain the maximum power, calculated as *P_L_* = *V_P,rms_*^2^/*R_L_*.

**Figure 9:**
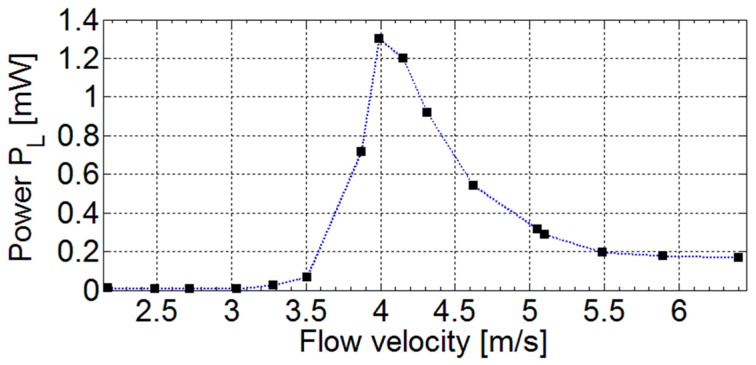
Measured power *P_L_* on a resistive load *R_L_* = 15 kΩ as a function of the flow velocity with the beam oriented at the angle *θ* = 300°.

The measured power as a function of the flow velocity with the beam positioned at the orientation angle *θ* = 300° is shown in Figure 9. The power extracted from the system presents a cut-in velocity of the airflow of about 3 m/s. The largest power values, up to 1.3 mW, are obtained for intermediate velocities where the vortex repetition frequency is close to the beam resonant frequency. For higher velocities, the power tends to a constant value of about 0.2 mW. This trend to constancy can be ascribed to the combination of two opposite contributions occurring for increasing flow velocity and vortex repetition frequency in turn. There is a positive contribution due to the increasing magnitude of the forcing, and there is a negative contribution due to the decreasing response of the beam excited out of resonance by the increasing repetition frequency.

From the measured power on the optimal resistive load an estimation of the performances in terms of power density and conversion efficiency can be obtained for the proposed harvesting system. Considering the area of the piezoelectric converter plus the blade, a maximum power per unit area of about 40 μW/cm^2^ is achieved. In addition, considering as the volume of the system the total region, comprising the bluff body, the beam with the converter, and the intermediate portion where the vortices arise, a maximum power density of about 2 μW/cm^3^ can be calculated. The obtained power density and power per unit area are comparable to similar reported harvesting systems [24,26]. The conversion efficiency can be defined as the ratio of the output electrical power to the input available power associated to the airflow which hits the exposed surface of the bluff body. As stated by the Betz law, only a portion *c_P_* ≈ 0.6 of the impinging power can be extracted from a mechanical-to-electrical converter. This portion goes down to *c_P_* ≈ 0.3–0.4 for reduced dimensions of the converter, as in the case of the proposed system [24]. Thus, the maximum electrical power as a function of the flow velocity can be calculated as:

(4) $$P_{el\_max}=c_{P}\frac{1}{2}\rho_{air}Su^{3}$$

where *S* is the exposed surface of the bluff body and *ρ_air_* is the air density. The proposed system presents an efficiency *P_L_/P_el_max_* of up to about 1.7% for the flow velocity *u* = 4 m/s where the maximum power is extracted.

## 3. Autonomous Sensors

The ability of the developed energy harvesting system to power autonomous sensors has been investigated by connecting the converter to a tailored electronic energy management unit plus a signal conditioning circuit and two sensors [27,28,29].

**Figure 10:**
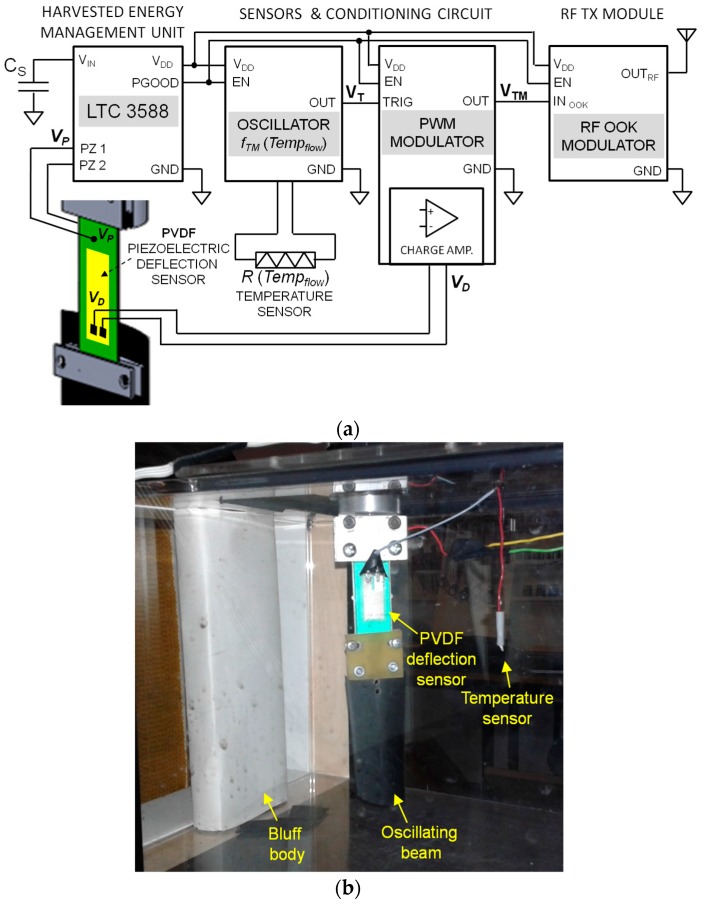
Block diagram of the autonomous sensor module powered by the piezoelectric converter (**a**); Picture of the realized system with the oscillating beam and the sensors placed behind the bluff body in the wind tunnel (**b**).

As shown in the block diagram of Figure 10, the energy management unit stores the harvested energy in a capacitor and periodically delivers it to the sensors and conditioning circuit and a RF transmission module. Once enough energy has been stored in the capacitor *C_S_* the management unit momentarily powers the conditioning circuit. This is based on a relaxation oscillator and PWM (pulse width modulator) circuit that together generate a rectangular-wave voltage *V_TM_*. The frequency of *V_TM_* depends on the flow temperature measured by a resistive temperature sensor in the flow, while the duty cycle of *V_TM_* is modulated by the voltage from an additional piezoelectric sensor placed onto the cantilever to sense the deflections. Therefore, the single waveform *V_TM_* simultaneously and independently carries in an energy efficient way information on both the flow temperature and the beam oscillations. The voltage *V_TM_* is then used as the control signal of a RF OOK (On-Off Keying) modulator to transmit the information on a 315 MHz carrier to an external receiving unit.

**Figure 11:**
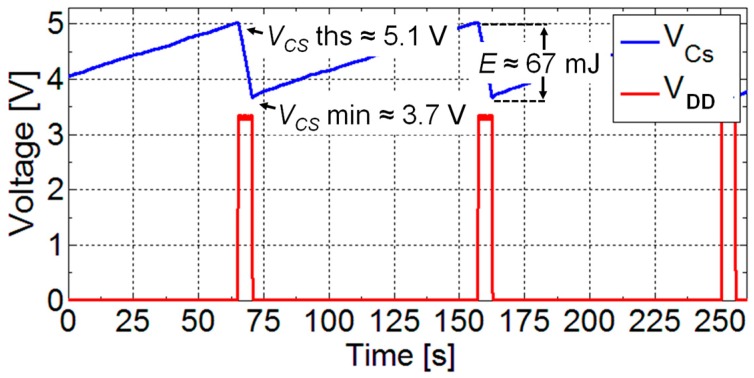
Operation cycle of the power management unit. The voltages V*_CS_THS_* and V*_CS_MIN_* on the storage capacitor represent the thresholds at which the load circuit is powered and disconnected, respectively.

The energy management unit is based on a dedicated integrated circuit (LTC 3588, Linear Technology, Milpitas, CA, USA) set to supply with a regulated voltage *V_DD_* = 3.3 V the load circuit. The input stage of the energy management unit is used to convert the AC voltage provided by the piezoelectric converter into a DC voltage used to charge the storage capacitor. Figure 11 reports a typical operation cycle of the unit. As it can be observed, when the voltage *V_CS_* on the storage capacitor *C_S_* is in the range that ensures the output regulated voltage, the load circuit is powered. In this way, the unit operation consists in charging phases followed by phases where the sensors, conditioning circuits and the RF transmission module are powered. During the charging phase, the harvested energy is stored in the capacitor *C_S_*. The following phase starts only when the voltage across the storage capacitor reaches the threshold voltage *V_CS_THS_* and thus enough energy to power sensors, conditioning circuits and to wireless transmit the information. An energy amount of about 67 mJ is stored and subsequently released in the alternating phases.

**Figure 12:**
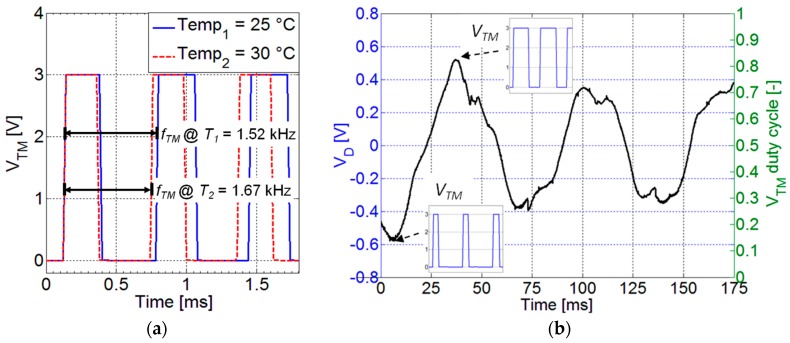
*V_TM_* for two different temperature of the flow (**a**); Example of the *V_D_* signal from the PVDF foil on the beam, and the associated duty cycle of *V_TM_* (**b**).

The generation of the signal *V_TM_* starts from a pulse wave generator which produces a signal *V_T_* with a frequency *f_TM_* dependent on the resistance of an NTC K164/10k temperature sensor (Epcos AG, Munich, Germany) placed in the flow. To this purpose the signal *V_T_* is used as the trigger for a pulse width modulator which generates the signal *V_TM_*. The duty cycle *DC_TM_* of *V_TM_* is controlled by the voltage *V_D_* generated by a PVDF piezoelectric foil that is attached onto the cantilever beam and acts as deflection sensor. Therefore, with reference to Figure 12a, *V_TM_* can be seen as a sequence of pulses with frequency *f_TM_* and duty cycle *DC_TM_* given by:

(5) $$f_{TM}=\frac{1}{T_{TM}}\approx f_{TM0}+K_{T}\left(Temp_{flow}-Temp_{0}\right)$$

(6) $$DC_{TM}=\frac{T_{ON}}{T_{TM}}=\frac{V_{D}-V_{D,min}}{V_{D,max}-V_{D,min}}$$

where *Temp_flow_* is the flow temperature and *Temp*_0_ = 25 °C is the reference ambient temperature; the frequency *f_TM_*_0_ is the base frequency at *Temp*_0_ and *K_T_* is the temperature sensitivity of *f_TM_*, the voltages *V_D,max_* and *V_D,min_* are the maximum and minimum values of *V_D_* corresponding to the maximum and minimum beam deflections. The linear relationship between frequency and temperature in Equation (5) results from the combination of the NTC sensor characteristic curve and the resistance-to-frequency conversion function of the relaxation oscillator [27]. As shown in Figure 12a, the temperature sensitivity *K_T_* of frequency *f_TM_* derived experimentally is 30.3 Hz/°C.

According to Equation (6), Figure 12b shows that *V_TM_* on its duty cycle *DC_TM_* carries information on the normalized value of *V_D_*.

An external receiver unit can reconstruct the rectangular wave *V_TM_* from the RF signal, extract the frequency *f_TM_* and duty cycle *DC_TM_* and, in turn, derive the flow temperature and the beam oscillation level, respectively. By analyzing the latter in the frequency domain as discussed in Section 2.3, the repetition frequency of the forcing vortices can be derived. Therefore, by the known *St* number of the proposed configuration and Equation (1), the information on the flow velocity can be also extracted [30].

**Figure 13:**
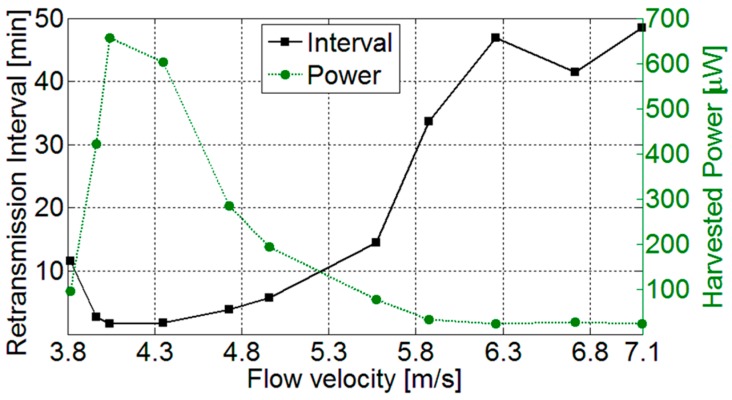
Average harvested power and retransmission time interval as a function of the flow velocity.

The performances of the harvesting system and energy management circuit have been characterized by measuring the transmission time and the time interval between consecutive transmissions as a function of the flow velocity. Considering a power consumption of the conditioning circuit and the RF transmission module of about 25 mW a transmission time duration of about 2 s has been obtained. In Figure 13 the measured retransmission intervals and the corresponding average harvested power are reported. Power values of up to 650 μW have been obtained and correspondingly a retransmission interval below 2 min has been achieved. As it can be observed, best performances are obtained, as expected, for flow velocities of about 4 m/s that produce vortex repetition frequencies that excite the beam resonance.

## 4. Conclusions

In this work the possibility of using a piezoelectric converter to harvest energy from airflow as a power source for autonomous sensors has been demonstrated. The pulsed force for the vibration of the piezoelectric beam converter is provided by von Karman vortices generated behind a bluff body placed in the airflow. A way to exploit the harvested energy to power autonomous sensors has been shown. Two sensors measuring the fluid temperature and flow velocity, and the respective signal conditioning circuits have been powered by a tailored energy management unit.

An analysis by CFD simulations has been carried out to demonstrate the effectiveness of the adopted configuration in generating an alternate velocity field. The theoretically expected linear trend between the inlet flow velocity and the vortices shedding frequency was confirmed by the numerical simulations.

Experimental results obtained varying the flow velocity in the explored range show that specific orientations of the beam converter behind the bluff body ensure better conversion performances. In addition, it is shown that the optimal harvesting effectiveness is obtained when the vortex repetition frequency is close to the resonant frequency of the beam. In this condition the system is able to generate a harvested power of about 1.3 mW on adapted resistive load for a flow velocity around 4 m/s. The system presents a cut-in velocity and an optimal velocity range followed by a reduction of the generated power for the higher velocities. Nevertheless, the experimental results show that the system is able to convert sufficient amount of energy and to power autonomous sensors also for flow velocity above the optimal working range, due to a careful design of the power management unit and of the low-power front-end circuits.

An improvement of the system to extend the operational bandwidth obtaining a wider optimal velocity range could be the use of a combination of multiple converter beams behind the bluff body exploiting both proximity effects [3,14] and different resonant frequencies.

The simultaneous measurement of air temperature and airflow velocity through an energetically autonomous module as offered by the present system represent an important capability in a wide range of applications in the industrial, building automation, automotive fields to name a few. The obtained results show that the proposed approach is viable for applications where battery-less maintenance-free sensor units and wireless transmission of flow information are required, for example to realize self-powered units in a WSN.

## Figures and Tables

**Table 1:** Grid convergence analysis: vortex frequency *f_u_* computed on the three grids, the order of convergence *p*, the GCIs and their relation are reported.

*u* (m/s)	*f_u_* (Hz) *h*_3_ = 4 mm	*f_u_* (Hz) *h*_2_ = 2.83 mm	*f_u_* (Hz) *h*_1_ = 2 mm	*p* (-)	GCI_2,3_ (%)	GCI_1,2_ (%)	$\frac{{\mathrm{GCI}}_{2,3}}{r^{p}{\mathrm{GCI}}_{1,2}}$ (-)
2.0	8.03	8.41	8.55	2.99	7.45	2.60	1.016
7.0	28.07	29.30	29.74	2.98	6.98	2.44	1.015
